# Climate Change Risk Perception in Taiwan: Correlation with Individual and Societal Factors

**DOI:** 10.3390/ijerph15010091

**Published:** 2018-01-08

**Authors:** Yingying Sun, Ziqiang Han

**Affiliations:** 1 Institute for Disaster Management and Reconstruction, Sichuan University, Chengdu 610200, China; sunying@scu.edu.com; 2 Center for Crisis Management Research, Tsinghua University, Beijing 100084, China

**Keywords:** risk perception, climate change, typhoon, individual factor, societal factor

## Abstract

This study differentiates the risk perception and influencing factors of climate change along the dimensions of global severity and personal threat. Using the 2013 Taiwan Social Change Survey (TSGS) data (N = 2001) as a representative sample of adults from Taiwan, we investigated the influencing factors of the risk perceptions of climate change in these two dimensions (global severity and personal threat). Logistic regression models were used to examine the correlations of individual factors (gender, age, education, climate-related disaster experience and risk awareness, marital status, employment status, household income, and perceived social status) and societal factors (religion, organizational embeddedness, and political affiliations) with the above two dimensions. The results demonstrate that climate-related disaster experience has no significant impact on either the perception of global severity or the perception of personal impact. However, climate-related risk awareness (regarding typhoons, in particular) is positively associated with both dimensions of the perceived risks of climate change. With higher education, individuals are more concerned about global severity than personal threat. Regarding societal factors, the supporters of political parties have higher risk perceptions of climate change than people who have no party affiliation. Religious believers have higher risk perceptions of personal threat than non-religious people. This paper ends with a discussion about the effectiveness of efforts to enhance risk perception of climate change with regard to global severity and personal threat.

## 1. Introduction

Climate change has become one of the most pressing global threats, especially for regions and countries that are located close to oceans, and affects human health in many ways, most of which are adverse. Thermal stress, extreme weather events, infectious diseases, future food security and prevalence of hunger, and other health risks caused by the social, demographic and economic distortions of climate change are the leading public health concerns related to climate change [1]. A recent review from the Lancet stated that the delayed response to climate change might jeopardize human life and livelihood globally, and so, under the umbrella of public health, there is a strong and urgent need for more studies on climate change [2].

To individuals, climate change is a vague term covering the increase in greenhouse gases, the rise of global temperatures, rising sea levels, melting ice sheets and glaciers, and increasing incidences of extreme weather events [3]. An investigation using lessons from more than 20 countries indicates that climate change has played an essential role in episodes of extreme weather [4]. Though there are still doubts regarding the contribution of human actions to climate change [5], rigorous scientific research has led most scientists to agree that human activities are the primary contributors to climate change [5,6,7,8,9]. Thus, besides the efforts of governments and public institutions [10], increasing the public’s awareness of climate change and encouraging the public’s adoption of climate-friendly behaviors are critical for mitigating the trends of climate change.

Human beings have two fundamental ways of comprehending risks: risk as analysis and risk as feeling [11]. In modern risk society [12], the concept of risk is seen as a consequence of modern development [13] with cultural variations [14,15]. Due to social amplification effects, risks can be amplified in the mass communication process, and influence individuals’ perceptions and decision-making [16]. The scientific characteristics of risks, such as dread, familiarity and uncertainty [17]; the informational factors, such as trust in sources; the personal factors, such as socioeconomic and demographic variables, as well as trust in authorities; and the contextual factors are the main influencing factors of risk perception, especially of natural hazards [18]. For technical risks, the balance between risks and benefits would affect the public’s attitudes. Taking nuclear power as an example, residents in the UK have to balance the potential threats of nuclear power plants and climate change, then finally make decisions according to their perceptions of the risks and benefits [19].

For climate change in particular, the previous literature reveals that attitudes toward climate change are usually related to gender, age, marital status, past experiences of disasters, levels of education, and levels of household income [20,21,22,23]. Studies have consistently documented that women tend to have higher risk perception than do men for a wide range of hazards [24] and are more likely to take measures for preparedness [23]. Recent surveys in the US have shown that younger adults hold stronger pro-environmental attitudes than do seniors and believe that the consequences of global warming should be taken more seriously [25]. Being married has been proven to be a significant predictor of risk perception and pro-environmental attitudes, and is associated with more actions [26]. Other studies of the perceptions of individual environmental risks, however, have found weak or non-existent trends concerning gender and age [27].

People’s experiences of extreme weather events provide a potentially significant route to increasing their awareness and encouraging their adoption of climate-friendly actions [28,29]. Personal exposure may help to anchor people’s understanding of climate change by making the potential risks more concrete and familiar [30]. The linkages between risk perceptions and behaviors are clear and supported by prior studies [31,32,33,34]. For example, research in China has shown that an improvement in the public’s perception of climate change may increase the desirability of adaptive behaviors, such as water conservation [35]. However, people may only increase their engagement with climate change mitigation if they attribute their experiences of extreme weather to climate change. Otherwise, they may simply ignore the threat or deny the existence of climate change [22].

Level of education can increase people’s perceptions of climate change. Individuals with higher levels of education tend to be more attentive to and knowledgeable about climate change [36,37,38]. Interestingly, a considerable number of studies have reported no significant association between education and adaptive actions [39,40].

Socioeconomic factors largely determine a household’s capability to take measures for preparedness [41]. Measures for commercial preparedness, such as purchasing flood insurance or strengthening buildings, require financial investments. Phillips et al. found that people in the lowest income quartile report greater restrictions on physical abilities, fewer community contacts, a higher concern for local hazards, and limited resources for taking protective actions [42]. However, when protective measures, such as storing food or having a family emergency plan, do not require much financial investment, income does not play a particularly important role in determining the adoption of protective actions [23].

Societal factors, which include politics, culture, religion, economic development, and technological innovation, may also influence people’s perceptions of climate change [43,44,45]. Cultural environmental biases are found to directly affect pro-environmental behaviors in the form of carbon-relevant household activities, such as conserving energy and water [24]. To encourage individuals to reduce carbon emissions, the cultural dimensions of climate change must be recognized [46]. Taiwanese society is traditionally characterized by the strong values and symbols of Confucianism and collectivism [47]. Reports have stated that most of the temples and churches, regardless of religious affiliation, in all 368 towns in Taiwan have a statue of Confucius [48]. These cultural and religious beliefs may have profound influences on an individual’s behavior. The impacts of ideological beliefs (as a specific religion) on climate change have attracted more attention in recent studies [49], though few have paid attention to the different influences of Eastern and Western religions.

Peer influences or social networks may also be important resources of information that affect an individual’s beliefs about climate change [50]. Prior studies indicate that the presence of links among distinct groups, relationships among individuals who share social identities, or networks of trust across authority gradients may lead to an increase in disaster risk perceptions [51,52]. However, studies do not always support such correlations. Taking heat waves as an example, one study from the UK indicates that strong bonding social networks could potentially exacerbate rather than reduce the vulnerability of elder people, because most of them did not view heat waves as significant risks [53]. Political orientation is another crucial societal factor that would affect people’s beliefs about and attitudes toward climate change, though the effects may vary in different countries. For example, political orientations, such as right-wing or left-wing, play a more relevant and critical role in shaping the public’s beliefs about anthropogenic climate change in the United States than in Germany or China [5].

Taiwan is an ideal place to examine the public perception of climate change because it is facing serious climate change issues [54] and climate change-induced threats of disasters. Taiwan is situated in one of the main paths of tropical cyclones in the Pacific Ocean and has experienced a series of typhoons with extraordinary amounts of rainfall and intense winds. Between 1958 and 2015, Taiwan experienced an average of 4.88 typhoon strikes per year [55,56]. Climate change is considered to be playing an important role in the increase of typhoons [57,58]. The increasing trend of climate change-related weather is a topic of policy debates [41]. The Taiwanese government has made a commitment to mitigate climate change [59], but the cooperation and coping of the public have yet to show any serious adaptations. Most of the current studies on climate change in Taiwan focus on the “hard” science aspect or the potential impacts, such as on water resources [60], the fishing industry [61], and infectious diseases [62], of climate change. An understanding of the public’s perception of climate change and the communication of information about climate change to the public are critical [9,43]. However, other than a study with a tiny sample, we have not found any significant investigations of this topic [63]. Our analysis uses an updated (2013), representative sample from Taiwan to investigate the public’s perception of climate change.

This paper is meant to fill in some of the gaps in current studies on climate change by examining the different roles of individual and societal factors in shaping public perception of climate change. Taiwan, a place with a strong Eastern culture facing high risks of climate change, is used to explore the perceptions of climate change and the influencing factors. Specifically, we (1) differentiate the perceptions of risks from climate change into two dimensions: global severity and personal threat; (2) analyze the roles of gender, age, education, experiences of climate-related disaster and risk awareness (anxiety), marital status, employment status, household income, and perceived social status in shaping risk perception of climate change; and finally, (3) investigate the impacts of social networks, including religion, political affiliations, and organizational embeddedness, on risk perceptions.

## 2. Methods

### 2.1. Survey Designs

The data used in this paper is from the 2013 Taiwan Social Change Survey (TSGS), which is a representative annual survey project in Taiwan. It is an official member of the International Social Survey Programme, which is a cross-national collaboration programme conducting annual surveys on topics relevant to social sciences across nations and regions worldwide [64]. The 2013 TSGS was designed by the Institute of Sociology, Academia Sinica, and the data collection was implemented by 48 trained interviewers from June to December 2013. The targeted respondents were adults older than 18 years of age in Taiwan. A three-stage stratified probability proportional (PPS) to the size sampling method was adopted in the survey. The primary sampling unit was the township, the secondary was the village, and the third was individuals. Detailed technical descriptions of this survey can be found in the technical manual and codebook of the survey [65]. A total of 2005 adults responded to the survey, and 2001 respondents were used in the analysis.

### 2.2. Measurements

#### 2.2.1. Dependent Variables

Two dimensions, global severity and personal threat, of risk perceptions of climate change were measured. For the first dimension, survey participants were asked, “Do you think climate change is a serious problem for the whole world?” For the second dimension, they were asked, “How much anxiety do you have about the potential impact of climate change on you and your family?” The answers to both questions were measured by a five-point Likert scale, but were recoded as binary variables for analysis because the data distribution violated the parallel assumptions [66,67]. For global severity, the answers of “not severe at all, not so severe, not clear” were coded as “0”, whereas the choices of “somewhat severe, very severe” were coded as “1”. Similarly, for personal threat, the answers of “no anxiety at all, low anxiety, difficult to say” were coded as “0”, whereas “some anxiety, high anxiety” were coded as “1”.

#### 2.2.2. Explanatory Variables

Climate-related disaster experience was a dummy variable indicating experiences of typhoons or inundations since 1993. The survey questions for this variable were, “How many times have you experienced typhoons/inundations since 1993?” and “How many times have you experienced inundations due to typhoons or heavy rainfall since 1993?”. If a respondent provided positive answers (non-zero) to either of the two questions, the variable was coded as “1”. Otherwise, it was coded as “0”. Typhoons and inundations are common extreme weather experiences that are strongly related to climate change [68]. Another variable related to climate change was anxiety about typhoons, which was measured by a five-point Likert scale ranging from “1” (very low) to “5” (very high).

Education, employment status, household income, perceived social status, marital status, gender, and age were the primary individual variables. Education was measured with a categorical variable from “1” to “4”: primary school or illiterate (1), middle school (2), high school (3), and tertiary education (4). Employment status was measured as “full-time, part-time, unemployed, student, and domestic worker”. Monthly household income was measured with a 26-categorical variable from 0 to more than 1 million New Taiwan dollars (NTD). Perceived social status was surveyed by asking the question, “How high do you perceive your social status to be?” to which the answers were in 10 categorical degrees ranging from “1” (the lowest) to “10” (the highest). Marital status was clustered into “single, married, divorced, and widowed”. Gender was a dummy variable with “1” representing male and age was a continuous variable.

Religion, organizational embeddedness, and political affiliations were the three variables adopted for the societal factors influencing the risk perception of climate change. Religion was originally measured by various specific types and coded into three categories: the non-believers (no religious affiliation), believers in Eastern religions (Buddhism, Taoism, I-Kuan Tao, Islam, folk faith and other local religions), and believers in Western religions (Christian or Catholicism). Organizational embeddedness was measured with a dummy variable with “1” representing membership in at least one proposed club/group, including political groups, community management boards, social service groups, religious groups, entertainment clubs, and labor unions. Organizational embeddedness represents the public participation of a respondent and is linked to social networks. Here, we intended to probe the correlations between risk perception and social networks. The political affiliation was measured by voting behaviors in the 2012 election, which was about half a year before the survey. The answers were clustered into four categories: voted for DPP (Democratic Progressive Party), voted for KMT (Kuo Min Tang) or PFP (People First Party), did not vote (no party), and ineligible to vote.

### 2.3. Data Analysis Strategy

The two measures of outcome, (1) the perceived severity of global climate change (binary) and (2) the perceived potential of personal impact from climate change (binary), were used as the dependent variables. Logistic regression models were used to estimate the effects. We recoded the original five categorical measures of the two dependent variables into dummies because the data distribution violated the parallel line assumptions when we tested the assumptions using the Generalized Logistic Regression/Partial Proportional Odds Models [66,67]. The analysis was implemented by statistical software, Stata version 13.1 MP (StataCorp. LP., College Station, TX, USA).

## 3. Results

### 3.1. Descriptive Analysis

The distribution of variables can be found in Table 1. Of the total number (2005) of respondents, 2001 observations were included in the analysis. Of the respondents, 50.97% were male while 49.03% were female. The ages of all respondents ranged from 18 to 100 years old with an average value of 47.23 (SD = 17.20) years. Marital status showed that 28.29% were single, 60.72% were married, 4.65% were divorced, and the last 6.35% were widowed.

Regarding the risk perception of climate change, 87.31% of the respondents perceived climate change as a serious global issue while 70.36% were anxious that climate change would affect them and their families. Regarding the individual factors shaping risk perceptions of climate change, the mean value of anxiety about typhoons was 3.09 with a range of “1” to “5”. Of the respondents, 42.13% had experienced a typhoon or an inundation caused by a typhoon or heavy rainfall. Regarding educational status, 18.09% had completed only primary school or were illiterate, 10.14% had completed only middle school, 7.90% had completed only high school, and 63.87% had tertiary education. Regarding current employment status, 52.37% were employed full-time, 12.89% were employed part-time, 3.10% were unemployed, 7.00% were students, and the last 24.64% were domestic workers. The average monthly income of a respondent was 9.10 in a 26-level category, indicating an amount between 70,000 to 80,000 NTD, which is about 2324 to 2656 US dollars (USD). For the perceived social status, the respondents had defined themselves at a level of around 4.64 on average in a ten-level category.

For the societal factors that may influence risk perceptions of climate change, 19.54% of the respondents had no religious beliefs, 75.66% were believers in Eastern religions, and the other 4.80% held Western religious beliefs. Regarding organizational embeddedness, 39.78% of the respondents were involved in some groups or clubs. For political affiliation (affiliations to specific parties), 28.14% had no affiliation, 41.48% supported the KMT or PFP, and 24.04% supported the DPP while the last 6.35% were not eligible to vote.

### 3.2. Perceptions of Climate Change and Correlates

The influences of the individual and societal factors on the two dimensions (global severity and personal threat) of risk perceptions of climate change were estimated by logistic regression models. The results (odds ratios) are shown in Table 2. The number of samples used in the estimations was 2001. Varied technical diagnostic tests were conducted [69] and the models were found to fit well.

Anxiety about typhoons was positively associated with both the perceived global severity and personal impact of climate change. With one degree of increase in anxiety, the perceived likelihood of global severity increased by 21% while the perceived likelihood of personal impact increased by 41%. Although experiences of climate-related disasters, typhoons and inundations were positively correlated with both perceptions, the results were not statistically significant.

Education positively influenced the concerns of climate change along both dimensions. Also, the impact of education on the perception of global severity was stronger than on personal impact. Compared with the respondents who were illiterate or had completed only primary school, those having completed middle school were 201% more likely to perceive global severity while those having completed high school and those with tertiary education were 421% and 453% more likely, respectively. For the perceived personal impact of climate change, respondents having completed only middle and high school were 116% and 209% more likely, respectively, to perceive personal impact as compared to those who were illiterate or had completed only primary school, but those with tertiary education were only 161% more likely.

Older adults had a lower probability of reporting perceptions of climate change risk along both dimensions of global severity and personal impact. Males had a lower probability of perceiving personal threat. Married respondents had a slightly higher probability of perceiving personal threat than did single respondents. Other socio-demographic characteristics, such as employment, household income, and perceived social status, were not significantly associated with perceived global severity or personal threat.

For the influence of the societal factors, people with religious beliefs had a higher likelihood of perceiving climate change risks than those without religious beliefs, but only the effects on perceived personal threat were statistically significant. The positive impact of organizational embeddedness on risk perception was significant only along the dimension of global severity. Concerning political affiliation, voters for DPP were 55% and 65% more likely to perceive risks of global severity and personal impact, respectively, than did those who had not voted in the last elections held in 2012. The KMT or PFP supporters had a lower probability of perceived risks, but it was significant only along the dimension of personal impact. Respondents who were ineligible to vote had a much higher likelihood of reporting the global severity of climate change.

## 4. Discussion

By analyzing updated and representative survey data from Taiwan, which faces serious threats from climate change, we innovatively differentiated the risk perception of climate change into dimensions of global severity and personal impact, then explored the effects of varied individual and societal factors on these two dimensions.

Within the individual features, as expected, more highly educated individuals have a higher probability of risk perception regarding climate change. Since more knowledge can be assimilated when more years are spent on education, awareness of climate change is more enhanced [23]. More interestingly, the outcomes of the analysis show that the effects of education on the risk perception of the global severity of climate change are stronger than that of the perceived personal impact. This result can be explained by the famous “normalcy bias” [70], by which people underestimate the possible effects of a disaster on themselves. Other valuable findings are the likelihood of perceiving global severity continuously increasing with higher level of education while the personal threat dimension does not exhibit the same trend. Therefore, effective efforts, such as disaster education, are required to promote the risk perception of personal impacts [71].

As we expected, people perceive the potential impacts of climate change differently along the two dimensions. These differences require a critical explanation of why higher risk perceptions do not necessarily demonstrate a robust capability to cope with potential disasters [72,73]. Having higher risk perceptions of the global dimension does not necessarily link to higher risk perception at the individual level, which in turn cannot motivate individuals to adopt climate-friendly actions. Although, in this paper, we cannot trace the exact mechanisms underlying the effects, our main argument is as follows. In the absence of differentiating the risk perception of the global dimension from the personal dimension, discussions about the effectiveness of education on risk perception are unilateral and may guide decision-making bodies in the wrong direction. The previous literature has shown that plans of risk preparedness could be positively influenced by socially accepted standards or media campaigns [23,74]. Therefore, in order to understand how people represent objects and events mentally, more consideration should be given to the difference in the psychological distance between global severity and personal threat as regards climate change.

Our results demonstrate that having experienced climate-related disaster is not significantly associated with perceptions of the severity of climate change along global or personal dimensions. In other words, prior experiences of disasters do not seem to play a significant role in enhancing risk perceptions of climate change, since people who have been affected by a disaster may lack strong evidence to link a specific disaster to climate change [22]. However, anxiety about typhoons and risk perceptions of climate change are significantly and positively correlated along both global and personal dimensions. Since local images of climate change are usually composed of extreme weather events and in Taiwan, tropical typhoons occur from June to October [55], the Taiwanese people pay close attention to typhoon risks. Therefore, these results provide strong evidence for studies that take extreme weather events as the main phenomena in discussions of issues of climate change in the academic field of disaster studies [3,75,76,77].

Regarding the societal aspects, we find that public participation has a positive correlation with the risk perception of climate change. Religious believers have higher risk perceptions of personal threat than the non-religious, indicating that ideological sensitivities may be embodied in how to live harmoniously with nature. Moreover, Western religious believers have higher risk perceptions than do Eastern religious believers. Previous studies have also revealed some similar observations about the role of religions in shaping people’s risk perception and behaviors. For example, Mitchell found that southern Baptist clergies were significantly more concerned about hurricanes than were Lutheran clergies in coastal regions [78]. Similarly, conservative Protestants demonstrated an overwhelming concern for economics, even at the expense of the environment, than did the Judeo-Christians [79]. Although we cannot further explain here the exact mechanisms of ideological influence on risk perceptions, our main argument is that religion could fundamentally define an individual’s attitudes and risk perceptions.

Regarding political affiliations, supporters of specific parties have higher risk perceptions regarding climate change than people who have no party affiliations. Since casting ballots is an important channel for engaging in public participation, this result suggests that having concerns about public affairs contributes to higher risk perceptions. This outcome is in line with the previous literature, which highlights the role of the decisions of political elites on policies of climate change [41]. For organizational embeddedness, the results suggest that people with organizational affiliations have higher risk perceptions of climate change more along the global dimension than on the personal dimension. Different degrees of influence inside the organizations may have caused the discrepancy between the factors of religion and organizational embeddedness.

This paper contributes to the current understanding of disaster risk reduction in three important ways. First, we extend beyond the existing literature, which usually conflates the perceptions of the impact of climate change on global severity and personal threat [24,35]. Our study shows that the personal and global dimensions of perceptions of the severity of climate change are crucially different, and identifies the underlying mechanisms through which the personal dimension may contribute to the adoption of climate-friendly actions. To our knowledge of the literature on climate change, this identification has not yet been done. Second, we provide new insights into the underlying determinants of risk perception by differentiating between the individual and societal factors. By examining the detailed factors in each domain, we have shown that societal factors may have a tremendous influence on promoting risk perceptions. In other words, community individuals can be more motivated to pay attention to risk perceptions of climate change if the surrounding environment is full of such efforts. Third, considering the fact that there exist relatively few studies on the impact of religious factors on risk perceptions of climate change, the ideological analysis is even scarcer. This paper provides new empirical evidence of the deep-seated religious beliefs in the domain of risk perception of climate change.

There are two main limitations of this study. The first limitation is in the research design. Since we used secondary data, i.e., data designed and collected by other scholars, and since climate change is only one part of the data collection efforts, some climate change issues like rising sea levels were not included. Future studies with specific designs for risk perception and climate change could remedy this. The second limitation concerns the methodology. Like other studies that rely on data from cross-sectional questionnaire surveys, this study largely relied on individual subjective reports of risk perception of climate change. Perceptions may have been overestimated by the respondents due to social desirability bias. Moreover, due to the cross-sectional nature of the data, we are unable to make causal claims on the correlation between the proposed influencing factors and the risk perception of climate change, though we have chosen the most appropriate strategies for data analysis by considering the data structures and theories. Thus, future studies that include both subjective risk perception of climate change and objective measures should be conducted, and longitudinal studies are desirable to understand the causes and effects.

## 5. Conclusions

In sum, this study has shown that both individual and societal factors play important roles in shaping the risk perceptions of climate change. An individual’s perception of climate change should be differentiated into dimensions of global severity and personal threat. Climate-related disaster experience is not significantly associated with higher risk perceptions of climate change, but anxiety about typhoons does positively correlate with higher risk perceptions of climate change along both dimensions. With higher levels of education, individuals would have higher perceptions of climate change, though the patterns for global severity and personal impact would not be the same. Societal factors, such as religious beliefs, organizational embeddedness, and political orientation, can be representative of ideology which shapes people’s perceptions of climate change.

## Figures and Tables

**Table 1:** Descriptive analysis (N = 2001).

Variable	Mean	SD	Min	Max
Age (years)	47.23	17.20	18	100
Household income	9.10	4.38	1	26
Perceived social status	4.64	1.73	1	10
Anxiety about typhoons	3.09	1.34	1	5
Variable	Item		Freq.	Percent
Global severity	Yes (“1”)		1747	87.31
Personal threat	Yes (“1”)		1408	70.36
Gender	Male		1020	50.97
	Female		981	49.03
Climate-related experience	Yes (“1”)		843	42.13
Organizational embeddedness	Yes (“1”)		796	39.78
Marital status	Single		566	28.28
	Married		1215	60.72
	Divorced		93	4.65
	Widowed		127	6.35
Education	Primary school or illiterate		362	18.09
	Middle school		203	10.14
	High school		158	7.90
	Tertiary education		1278	63.87
Employment	Full-time		1048	52.37
	Part-time		258	12.89
	Unemployed		62	3.10
	Student		140	7.00
	Domestic work		493	24.64
Religion	No religion		391	19.54
	Eastern		1514	75.66
	Western		96	4.80
Political affiliation	No party		563	28.14
	KMT or PFP		830	41.47
	DPP		481	24.04
	Ineligible to vote		127	6.35
Total			2001	100

**Table 2:** Logistic regression on risk perception of climate change (N = 2001).

	Global Severity (Odds Ratio)	Personal Threat (Odds Ratio)
Gender	0.75 (0.55, 1.03)	0.58 *** (0.46, 0.72)
Age	0.98 ** (0.96, 0.99)	0.99 * (0.97, 1.00)
Marital status (Single as reference)		
Married	1.42 (0.89, 2.27)	1.64 ** (1.20, 2.25)
Divorced	1.84 (0.80, 4.23)	1.10 (0.64, 1.89)
Widowed	1.26 (0.64, 2.51)	1.32 (0.76, 2.27)
Anxiety of typhoon	1.21 *** (1.08, 1.35)	1.41 *** (1.30, 1.53)
Climate-related disaster experience	1.30 (0.95, 1.77)	1.00 (0.81, 1.24)
Education (Primary school or illiterate as reference)		
Middle School	3.01 *** (1.80, 5.05)	2.16 *** (1.41, 3.28)
High School	5.21 *** (2.63, 10.32)	3.09 *** (1.90, 5.05)
Tertiary Education	5.53 *** (3.49, 8.76)	2.61 *** (1.82, 3.74)
Employment (Full-time as reference)		
Part-time	1.18 (0.72, 1.94)	1.27 (0.90, 1.80)
Unemployed	0.33 ** (0.16, 0.67)	0.83 (0.47, 1.48)
Students	0.96 (0.34, 2.74)	1.07 (0.63, 1.82)
Domestic work	0.94 (0.61, 1.45)	0.95 (0.69, 1.32)
Household income	0.98 (0.94, 1.01)	0.98 (0.96, 1.01)
Perceived social status	0.94 (0.86, 1.02)	1.01 (0.95, 1.07)
Religion (No religion as reference)		
Eastern	1.26 (0.86, 1.86)	1.62 *** (1.26, 2.10)
Western	2.14 (0.83, 5.50)	2.33 ** (1.30, 4.16)
Organizational embeddedness	1.58 ** (1.16, 2.16)	1.17 (0.94, 1.45)
Political affiliation (No affiliation as reference)		
KMT or PFP	1.39 (0.99, 1.94)	1.38 * (1.07, 1.77)
DPP	1.55 * (1.04, 2.30)	1.65 *** (1.23, 2.20)
Ineligible to vote	5.59 * (1.21, 25.78)	1.33 (0.76, 2.31)
Pseudo-R2	0.159	0.089

Exponentiated coefficients; 95% confidence intervals in brackets; * *p* < 0.05, ** *p* < 0.01, *** *p* < 0.001.
